# Environmental Contaminants and Pancreatic Beta-Cells

**DOI:** 10.4274/jcrpe.2812

**Published:** 2016-09-01

**Authors:** Gabriel Fabricio, Ananda Malta, Abalo Chango, Paulo Cezar De Freitas Mathias

**Affiliations:** 1 CAPES-Foundation, Ministry of Education Brazil, Brasilia, Brazil; 2 State University of Maringá, Department of Cell Biology and Genetics, Laboratory of Secretion Cell Biology, Maringá, Brazil; 3 UPSP-EGEAL Polytechnic Institute LaSalle de Beauvais, Beauvais, France

**Keywords:** Contaminants, pancreatic beta-cell, Diabetes, insulin resistance

## Abstract

Despite health policies as well as clinical and research efforts, diabetes prevalence is still rising around the world. A multitude of causes have been suggested for this increase, mostly related to familial background, the occidental diet which is rich in fat/carbohydrates, and sedentary life style. Type 2 diabetes involves malfunctions of the primary pancreatic beta-cells, usually attributed to local damage; however, it can be associated with other stressful environmental agents, such as chemical contaminants from food, plastic and air, among others. Indeed, exposure to these chemical agents during perinatal and adolescent life can increase the risk of developing cardiometabolic diseases later in life. This review explores data showing which environmental chemical agents may produce injury in beta-cells and further impair the insulinotropic process of type 2 diabetes. Additionally, it points the need to also consider unusual causes of metabolic diseases, such as environmental contaminants.

## INTRODUCTION

Type 2 diabetes is becoming a great problem for world health authorities, and it is generally accepted that a sedentary lifestyle, an increase in high-fat diets, and genetic factors do not completely explain this epidemic, whose origin may be in the maternal womb (1). Among all the functions of the pancreas, insulin production by beta-cells has received great attention due to their functions related to glycemia control, which is an important hallmark of metabolism control. Beta-cells are very sensitive and are the only cell type that can produce, store, and release insulin; when they fail or are destroyed or affected, the metabolism is impaired and the onset of diabetes may occur (2). The endocrine pancreas is formed by pancreatic islets, also termed Langerhans islets, consisting of four different types of endocrine cells known as the beta, delta, alpha, and pancreatic polypeptide (PP) cells which produce, store, and release insulin, glucagon, somatostatin, and pancreatic peptide, respectively. Islets also occur in lower quantities in the acinar structures of the pancreas. Although beta-cells occupy a great part of the volume in the islets, their capacity to proliferate and their neo-formation are extremely reduced compared to other cells (3). Insulin secretion control is distinct from all other cells that release hormones or neurotransmitters; glucose, as a major physiological stimulus, induces very high cellular metabolism activity, promoting the production of adenosine triphosphate (ATP) which can block a specific potassium (K+) channel. Once K+ output is reduced, the beta-cell is depolarized which induces certain calcium (Ca^2+^) channels to open, permitting a rise in intracellular Ca^2+^ (1). The cytoskeleton is activated, stimulating the transport of vesicles that contain insulin to the periphery of plasma membrane, where exocytosis is performed and the insulin is released. Save for a few neurons in the hypothalamus, beta-cells can be considered a unique metabolic sensor. Other secretory cells are signalized using mostly a membrane receptor (4).

The high incidence of metabolic diseases such as type 2 diabetes cannot be explained solely by the occidental diet and sedentary life style. In the last 10 years, it has been shown that the influence of environmental pollutants such as food contaminants, plasticizers, pesticides (Ps), and organic metallic compounds might contribute to the early onset of obesity and beta-cell malfunction which allows the development of diabetes (5). Environmental contaminants injure pancreatic beta-cells impairing the metabolism; however, when the exposure is halted, the beta-cells might recover improving the metabolism. Nevertheless, exposure to contaminant during perinatal life can damage the beta-cells disrupting fetal metabolism, a state which can persist even in adulthood (1).

There is a theory that certain challenges during gestation, mostly malnourishment, including a restricted caloric diet, that stress the fetus in the maternal womb can compromise growth and development. In this condition, babies are delivered with low birth weight and are at high risk for cardiometabolic disorders such as obesity, hypertension, and diabetes (6). The stressed fetus adopts strategies to maximize its chances for survival and growth in postnatal life. Adapting its metabolism to live in restricted caloric conditions is one such strategy. These survival maneuvers promote the maintenance of noble tissues, such as the brain and heart, but can compromise that of others such as the liver, muscles, and pancreas. However, upon exposure to food abundance, the metabolism saves the excess and overaccumulates energy stocks. This concept, which emerged in the 1990s, is known as developmental origins of health and disease (DOHaD) but also termed the Barker theory, the thrifty phenotype theory, and metabolic programming, among others (7). Interestingly, in the 2000s, a theory that assigns environmental chemicals which affect adults as well as fetuses has emerged. According to this theory, environmental chemicals are the cause of the cardiometabolic disruption pandemic worldwide (8). During gestation, to maintain the development of the fetus, hormone and nutrient blood levels, including insulin, leptin, estrogen, and glucose, show an increase (1). Thus, the muscles and adipose tissues of the mother reduce their glucose uptake, building an insulin-resistant framework for a specific time (9). However, when insulin secretion/insulin sensitivity is impaired, an increase in insulin resistance is triggered, inducing gestational diabetes mellitus (GDM) which in turn is linked to high birth weight, early onset of obesity, and type 2 diabetes in adulthood (1).

Specifically the first and third parts of gestation are critical periods in the development of pancreatic islets in humans and rodents, respectively (2). The end of this process, whereby the function of the pancreatic islets are established, occurs during the lactation period (10). Regarding pollutants and precocious diabetes, there is strong evidence that environmental pollutants are present in the mother’s placenta and can be transferred to the fetus producing diabetes in later stages of life (8). However, the mechanisms by which pollutants might program the fetus to develop diabetes in adulthood are not yet clear or fully understood. This brief review aims to help understand the relationship between precocious exposure to environmental pollutants and pancreatic beta-cell damage and the possible role of this relationship in type 2 diabetes onset.

## FOOD CONTAMINANTS AND DIABETES

Chemicals or pollutants that are generally called endocrine-disrupting chemicals (EDCs) act directly on the function of endocrine system and may inhibit the release and action of several hormones related with body metabolism (11). Originally, these chemicals were thought to act primarily through nuclear hormone receptors, including estrogen receptors (ERs), androgen receptors (ARs), progesterone receptors, thyroid receptors (TRs), and retinoid receptors, among others, but with increasing knowledge, the mechanisms of action have been recognized as much broader, and an EDC is now accepted as any compound, either natural or synthetic, that, through environmental or inappropriate exposure, alters the hormonal system (12). EDCs include industrial contaminants, plasticizers, food contaminants, polychlorinated biphenyls (PCBs), metals, Ps, and other chemicals that can be accumulated in body tissues, mainly in adipose tissue due to their lipophilic nature (13). It is accepted that the EDCs, since they have a great affinity for ERs alpha and beta (ERα, ERβ), can impair the hormonal system mainly during gestation, leading to deregulation of functions related to sexual differentiation as well as affecting insulin sensitivity and production in the mother and the fetus (5).

As commented above, these compounds can deregulate body metabolism. However, it must also be said that EDCs act in different ways which vary by range of dose, time of exposition, and the particular metabolic pathway. In other words, an individual, following exposure to one of these compounds, may or may not show a response (14). The relevant literature indicates that in humans, the EDCs may produce biological changes in doses lower than the allowed lowest doses, producing U shaped or inverted-U shaped dose–response curves. These so-called nonmonotonic doses represent a response with a change in the sign to positive for negative or vice and versa in the slope over the dose range tested. The PCBs can act as an agonist or antagonist and inhibit the hypothalamic-pituitary-thyroid axis (HPA) or even lead to impairment of some TRs and affect thyroid hormone signaling and action (15). Although the EDCs are claimed to elicit obesity by acting directly on white adipose tissue, other structures such as brain, liver, the endocrine pancreas, and especially pancreatic beta-cells, may be direct targets as well (14). Taken together the above-presented evidences, the sections of this review aim to show that exposure to any type of EDC may possibly lead to changes in the structure or function of pancreatic beta-cells and play a role in type 2 diabetes onset.

## XENOESTROGENS AND ESTROGENS

During gestation, regardless of development of GDM, insulin resistance increases due to the requirement of the fetus for glucose (16). The increase of estrogen appears to be linked to insulin secretion and sensitivity as well as to GDM (5). In fact, several studies show that during pregnancy, the pancreatic islets adapt to the high insulin demand. It has been shown that the islets are high in size and number and that proliferation and neogenesis are dramatically increased in beta-cells during this period (17). In the past, this increase was attributed to lactogenic hormones; however, estrogen is currently associated with the increases in insulin secretion and sensitivity. This sexual hormone promotes a protective effect against oxidative stress and pro-inflammatory cytokine-induced apoptosis in pancreatic beta-cells (1).

In support of this point, it is known that low levels of estrogen in ovariectomized or aged rats are associated with glucose intolerance, insulin resistance, dyslipidemia, and obesity but, interestingly, it was shown that an excess of estrogen also induced metabolic disruption causing obesity (18). Beta-cells present ERs (ERα and ERβ) (1). Indeed, knockout mice to estrogen alpha-receptor (ERαKO) exhibit a reduction in glucose transporter type 4 (GLUT4), while knockout mice to estrogen beta-receptor (ERβKO) present no differences although it is well documented that ERβ is involved in body fat distribution (5). These observations and comments provide evidence that estrogen can influence beta-cell function, and unexpected estrogen fluctuation levels might contribute to metabolic diseases.

Within this context, it has been accepted that estrogens and xenoestrogens such as bisphenol A (BPA), a contaminant associated with plastic packs, may stimulate high production of estrogen in both females and males with substantial effects (19). In fact, estrogen elevation induces a rise in Ca^+2^ in beta-cells, due to closure of the K_atp_ channel, provoking an increase in insulin secretion (16). This same study also shows that not ERα but ERβ mediates rapid estradiol effect, and thus, in synergy with glucose, when the estradiol binds to ERβ, the guanylate cyclase receptor is activated through an unknown pathway. As a consequence, K_atp_ channel activity decreases in a cGMP-dependent protein kinase (cGMP/PKG) in a dependent manner, and this effect finally potentiates enhanced insulin secretion (20). Moreover, in rats that received anti-estrogens, the content of insulin was decreased (1). However, it is important to emphasize that estrogen causes insulin resistance, which in turn provokes insulin oversecretion. Estrogen is also associated directly with insulin release from beta-cells. Thus, estrogen leads by different pathways to beta-cell exhaustion, death, and inhibited proliferation, allowing the onset of type 2 diabetes (16).

## PERSISTENT ORGANIC POLLUTANTS

Regarding the growth of the type 2 diabetes epidemic, many studies have indicated the influence of persistent organic pollutants (POPs). Exposure to this type of pollutant during gestation and lactation causes glucose intolerance in the offspring, supporting the existence of a potential mechanism for triggering adulthood diabetes (21). POPs are highly spread in the environment and in high doses can provoke toxicity in animals and human beings. Mainly, this class of contaminants impairs neuroendocrine functions (22).

Among POPs, it is known that the contaminant 2,3,7,8-tetrachlorodibenzo-p-dioxin (TCDD), used as a herbicide (no more commercialized applications), is one of the most toxic. Its effects are exerted through the activation of a specific cytosolic receptor, the aryl hydrocarbon receptor (AhR) (21). TCDD exposure in animals is related to wasting syndrome, characterized by loss of body weight or reduced weight gain due to difficulty in using blood nutrients, a condition also associated with hyperglycemia (23). TCDD also reduces glucose uptake in the adipose tissue, pancreas, and liver (22).

Cell death due to an increase in pro-inflammation cytokines is observed in insulin secreting cells (INS-1E) when exposed to low doses of TCDD (12,5 and 25 nM) for only 1 hour (24). Thus, beta-cells may be an important target for the action of this contaminant. In fact, TCDD has been observed to stimulate autophagy in cultured cells (25). In advanced stages of type 2 diabetes, autophagy in beta-cells occurs due to increased inflammation signals contributing to the decrease in insulin secretion (26).

Another pathway for increased diabetes onset mediated by TCDD is TCDD-induced (Ca^+2^) influx via calcium T-type channels that regulate vesicular trafficking, such as lysosomal and secretory granule exocytosis. According to the increase in Ca^+2^ is linked to beta-cell depolarization and thus related to reduced K_atp_ channel activity and increased insulin secretion and the possible exhaustion of beta-cells (22).

## METALS

Diabetes is widely explained as a physiopathological situation involving difficulty in promoting glucose uptake by peripheral tissues, mainly due to impaired insulin secretion and/or low insulin sensitivity in the glucose uptake tissues (27). It has been accepted that with advancing age, this diabetic framework may worsen, and pollutants with long half-lives such as some metals may be involved in this progression (28).

Cadmium is a non-essential toxic metal that is highly present in the environment (29). Other than tobacco, diet is the major source of Cd and it is found in cereals, potatoes, and root vegetables. It has been identified as a metallo-estrogen (30). It has been observed that Cd gradually accumulates in beta-cells. Studies show that Cd has a half-life of 30 years. Indeed, long-term Cd exposure induces a reduction in insulin secretion (28). However, the mechanisms involved in beta-cell failure due to Cd exposure are not known. One study suggested reduced calcium uptake by the pancreatic beta-cells (31), but further studies are needed to determine the correct pathways behind Cd and type 2 diabetes.

In addition to Cd, arsenic (As) is also known as a potential stressor of pancreatic cells, and the main source of contact is contaminated water (32). It is known that As stimulates autophagy maneuvers due to increased reactive oxygen species (ROS). Zhu et al (33) showed that when insulin-secreting (INS-1) cells are exposed to low doses of As (1-4 µM) for 24 hours, the production of ROS was increased, which was related to cell death due to high autophagy and resulted in reduced insulin secretion. Alternatively, it has been shown that the transcription of insulin genes such as Pdx1, a gene involved in pancreatic cell maturation and survival, is downregulated by As exposure (34).

Last but not least, mercury (35) is also related to glucose intolerance (36). However, much of what is known about its toxic effects is related to nervous system development (37). The major source of Hg is seafood consumption (38). In relation to pancreatic beta-cell failure, it is reported that Hg in low doses can induce cell stress and cellular death and cause pancreatic islet beta-cell dysfunction which may lead to diabetes development (37,39). In rodents, a low dose of Hg was able to induce an increase in oxidative stress leading to signaling pathway activation of phosphatidylinositol 3-kinase (PI3K)-Akt, which was related to impaired insulin secretion (39).

However, not only these metals but, according to other studies, excess or deficiency of certain essential trace metals such as zinc and nickel may play an important role in beta-cell malfunction. In fact, available evidence shows that these elements are found in large quantities in diabetic persons, however, more studies are needed to show this relationship and the possible pathways involved (37).

## PESTICIDES

Exposure to Ps has been related to neurotoxicity, but there is a growing body of evidence indicating that Ps can induce metabolic diseases, such as obesity and type 2 diabetes (40). In fact, Ps are considered carcinogenic EDCs interacting with estrogens mainly via the thyroid hormones and increasing the expression of estrogen-responsive genes (41). Ps, mainly organophosphates, attack neuron connections via blocking acetylcholinesterase (AChE) activity but can also induce pancreatitis, thus damaging beta-cells. Moreover, hyperglycemia is frequently associated with exposure to Ps (40). Although little is known about how Ps can induce hyperglycemia, it is suggested that the inhibition of AChE activity may increase ROS and lead to high cell death (41).

Malathion, a specific pesticide, is used as an insecticide in agricultural, veterinary, medical, and public health practice (41). It has been associated with metabolic disorders such as obesity and diabetes (42). In relation to type 2 diabetes, malathion causes an increase of intracellular Ca^+2^ in beta-cells, which could cause a loss of Ca^+2^/calmodulin-dependent protein kinase function that is involved in the regulation of insulin secretion (41). Moreover, malathion is associated with increased apoptosis due to toxic effects on islet mitochondria thus increasing ROS production.

Another pesticide, diazinon, which is also employed in agricultural practice, is associated with impairment in glucose uptake and insulin secretion. Pakzad et al (42) suggested that the pathway of this metabolic disorder is mainly through the stimulation of muscarinic receptors which are involved in the process of glucose-induced insulin secretion. As an acetylcholine agonist, diazinon potentiates glucose-induced insulin secretion. High doses and/or long-term exposure to this pesticide might exhaust pancreatic beta-cells, allowing diabetes onset (42,43). In other words, exposure to Ps can result in increased AChE overstimulation, down regulation of muscarinic receptors, and most likely reduced production of insulin, similar to findings in rats exposed to a high-fat diet and mono sodium L-glutamate (43,44). In results not yet published, our laboratory has shown that perinatal exposure to acephate, another organophosphate pesticide, provokes beta-cell malfunction in the adult life of rat offspring. Collectively, all this information gives clear evidence associating pesticide exposure and type 2 diabetes, but further studies are needed for accurate determination of the pathways involved.

## FOOD PROCESSING PRODUCTS

It is known that environmental pollutants are linked to the increasing diabetes epidemic and that reactive species accepted as contaminants are produced in food storage or preparation. Examples include advanced glycation end products (AGEs) of the Maillard reaction (45). More specifically, the Maillard reaction is a reduction reaction of carbohydrates with amino compounds which is responsible for the aroma, taste, and appearance of thermally processed food (46). The best-known AGE products are methylglyoxal (MG), acrylamide (AA), and N(ε) -(carboxymethyl) lysine (CML) which in elevated levels are associated with glucose intolerance and insulin resistance (45).

Excess glucose has been accepted as a precursor of beta-cell damage receiving the name of glucotoxicity (47). In fact, this toxicity due to hyperglycemia, in the long term, can increase the levels of ROS and endoplasmic reticulum (ER) stress, which are related to high levels of Ca^+2^ and the occurrence of the glycation reaction (48). The glycation reaction has been linked to the irreversible and heterogeneous production of species or products from reactive dicarbonyls, in other words, the non-enzymatic linkage of glucose with an amino group; the products of this reaction are termed AGEs (47).

The main source of AGEs is an increase in intake of thermally processed foods. Sustained hyperglycemia is one other cause (glucotoxicity) (48). Glucotoxicity is identified as an important characteristic of problems related to beta-cell failure and type 2 diabetes because it contributes to increased ROS production, ER stress, and intracellular and extracellular AGEs formation (49). The action of AGEs is triggered by the linkage between an AGE and the receptor termed advanced glycation end products receptor (RAGE) (50). The AGE products overstimulate RAGE resulting in decreased glucose-stimulated insulin secretion and high cell apoptosis due to the elevation of ROS (48). Pancreatic islets isolated from normal adult rats with MG for 24 hours show an altered insulin secretion response to glucose, which suggests beta-cells as a target of AGE substances (51).

## WHAT IS THE IMPORTANCE OF ENDOCRINE DISRUPTING CHEMICAL MIXTURES?

In the previous sections, information was given about the role of different contaminants in the progression of the diabetes type 2 epidemic, and the need to focus efforts on developing therapies for reducing this framework was brought to attention. However, it is important to emphasize that the individual is in constant lifelong contact with the environment, continuously receiving different stimuli. Thus, it is likely that many EDCs are acting together because environmental contamination is rarely due to a single compound, and the effects of different classes of EDCs may be additive or even synergistic (12).

Normally, humans or animals are exposed to a great variety of known and unknown contaminants throughout life, and individuals have differences in metabolism, body composition, and gene expression that can increase or decrease the half-life of pollutants, which may or may not produce their potential effects (52). Regarding metabolic disorders, it is likely that the progression of these diseases is the result of chronic exposure to mixtures of low amounts of EDCs, and the latency between pollutant exposure and clinical disorders creates further challenges in attempting to establish a relationship to the level of exposure and the physiology of each person (12).

## METABOLIC PROGRAMMING AND FOOD CONTAMINANTS

Regarding the formation of pancreatic islets in rodents, it is known that at 14 days of gestation, in response to the influence of different growth factors, hitherto non-differentiated stem cells promote the emergence of different cell types representing a precocious maturing stage of pancreatic islets. In humans, the same process occurs in the first trimester of gestation, more specifically in the first ten weeks of life (53,54). In rodents, the immature pancreas presents a few cells that produce glucagon, which appears to be responsible for stimulating the initial production of insulin-secreting cells; however, this stage does not yet represent the true insulin secretion. In humans, insulin secretion begins during gestation, but in rats, it occurs only during lactation (53,55). Within the pancreatic islets formation process, several transcription factors are indispensable for promoting maturation and defining the future function of the cells. The expression of many transcription factors, such as paired box Pax4 and 6, homeodomain (Nkx) 6.1 and 2.2, fork head box (Fox) O1 and A2, neurogenin (Ngn) 3, and pancreatic and duodenal homeobox-1 (Pdx-1), among others, contribute as crucial markers in the pancreatic beta-cells for growth and survival throughout life (27).

Among all the functions of insulin, the most essential is ensuring that sugar is correctly taken up and stored in peripheral tissues such as the liver, muscles, and adipose tissue (13). Adequate glucose sensitivity, beyond all other nutrients, rapidly regulates insulin synthesis and secretion and is also critical for the maintenance of the glucose-responsive state and the number of beta-cells (40). During the development of the pancreas as well as subsequently, beta-cell apoptosis is normal, although this loss is compensated by neogenesis from preexisting beta-cells or from trans-differentiation of acinar cells (56). Thus, it is important to emphasize that outside the period during which the pancreas is formed, the amount of beta-cells may increase or decrease, and this process is dependent on how beta-cell formation occurred, in other words, if it was stressed or not (57).

Regarding precocious exposure to EDCs, since the studies of Barker about thrifty phenotype, more recent studies regarding another theory following the same principle have shown that early nutritional insults combined with exposure to environmental pollutants promoted an increase in obesity and related diseases and may have an influence on the formation of the pancreatic islets. In fact, it is accepted that during fetal life, the individual is more susceptible to environmental insults and these insults may lead to irreversible gene expression alterations (13,58).

Metabolic programming develops epigenetic modulation, which is defined as heritable changes in gene expression that are not due to any alteration in the primary DNA sequence. Epigenetic mechanisms include DNA methylation, histone modification, and regulation by noncoding RNAs. The way the genome interacts with and responds to the environment and even potentially the way the genome can influence its own environment via its effects on behavior are controlled by epigenetic changes (59). Epigenetic changes, particularly in DNA methylation, provide a “memory” of developmental plastic responses to the early environment and are central to the generation of phenotypes and their stability throughout life (13). As shown in this review, it is known that when estrogen action occurs at an inappropriate time or at non-physiological levels, adverse effects such as insulin resistance and hyperglycemia may occur. In addition, when glucose tolerance is impaired during pregnancy, the levels of DNA methylation in the genes involved in islet development may be modified, allowing reduced expression; in fact, the metabolism of early embryos is affected by maternal hyperglycemia, which causes the down-regulation of embryonic genes related to insulin action, such as GLUT 1,2, and 3 in the blastocyst stage (60).

In accordance with these points, Lin et al (61) showed that maternal exposure to Bis(2-ethylhexyl) phthalate (DEHP) promotes the reduction of important genes involved in beta-cell development. The authors showed that Pdx-1, a gene involved in the origin of pancreatic islets formation, was downregulated in the offspring of mothers exposed to DEHP during gestation. Pdx-1 plays an important role in islet formation and gene transcription, and thus the reduction or even absence of this gene impairs insulin production and insulin output. In other words, the reduction in beta-cells provoked by DEHP during perinatal life, through decreased Pdx-1 gene expression, compromises the beta-cell function of the offspring (61). Moreover, downregulated Pdx-1 is also related to impaired mitochondrial function (62). During pancreas formation, beta-cells have high energy demand and poor antioxidant defense, so when mitochondrial function is decreased, an increase in ROS occurs promoting damage to mitochondrial DNA that is associated with the emergence of diabetes in adulthood (55).

To conclude, it is widely accepted that the fetal period is critical for the development of healthy beta-cells in adulthood. The food intake behavior and environment of the mother may program the child for future health and unfortunately, future diseases. Not all pathways for this malprogramming are known, but new knowledge regarding the relation between contaminants and diseases can greatly aid in eliminating the metabolic pandemic, saving future lives.

## Ethics

Peer-review: Externally peer-reviewed.
